# Design and validation of cyanobacteria-rhizobacteria consortia for tomato seedlings growth promotion

**DOI:** 10.1038/s41598-022-17547-8

**Published:** 2022-07-31

**Authors:** A. J. Toribio, F. Suárez-Estrella, M. M. Jurado, J. A. López-González, M. R. Martínez-Gallardo, M. J. López

**Affiliations:** Department of Biology and Geology, CITE II-B, University of Almería, Agrifood Campus of International Excellence, ceiA3, CIAIMBITAL, 04120 Almeria, Spain

**Keywords:** Applied microbiology, Bacterial techniques and applications

## Abstract

The use of rhizobacteria provide great benefits in terms of nitrogen supply, suppression of plant diseases, or production of vitamins and phytohormones that stimulate the plant growth. At the same time, cyanobacteria can photosynthesize, fix nitrogen, synthesize substances that stimulate rhizogenesis, plant aerial growth, or even suppose an extra supply of carbon usable by heterotrophic bacteria, as well as act as biological control agents, give them an enormous value as plant growth promoters. The present study focused on the in vitro establishment of consortia using heterotrophic bacteria and cyanobacteria and the determination of their effectiveness in the development of tomato seedlings. Microbial collection was composed of 3 cyanobacteria (SAB-M612 and SAB-B866 belonging to *Nostocaceae* Family) and GS (unidentified cyanobacterium) and two phosphate and potassium solubilizing heterotrophic bacteria (*Pseudomonas putida*-BIO175 and *Pantoea cypripedii*-BIO175). The results revealed the influence of the culture medium, incubation time and the microbial components of each consortium in determining their success as biofertilizers. In this work, the most compatible consortia were obtained by combining the SAB-B866 and GS cyanobacteria with either of the two heterotrophic bacteria. Cyanobacteria GS promoted the growth of both rhizobacteria in vitro (increasing logarithmic units when they grew together). While Cyanobacteria SAB-B866 together with both rhizobacteria stimulated the growth of tomato seedlings *in planta*, leading to greater aerial development of the treated seedlings. Parameters such as fresh weight and stem diameter stood out in the plants treated with the consortia (SAB-B866 and both bacteria) compared to the untreated plants, where the values doubled. However, the increase was more discrete for the parameters stem length and number of leaves. These results suggest that the artificial formulation of microbial consortia can have positive synergistic effects on plant growth, which is of enormous agro-biotechnological interest.

## Introduction

Plants are extensively colonized by a range of beneficial microorganisms with roles as plant growth promoters and biopesticides^1,2^. These microorganisms are involved in a set of interactions known to affect plant health and soil quality. They also participate in activities that ensure the stability and productivity of both agricultural systems and natural ecosystems. Previous studies have shown that cooperation between different microorganisms can be exploited as a beneficial activity. There are examples of this related to cultivation, and production of plants in an environmentally friendly way^3^. Because of current public concerns about the side-effects of agrochemicals, there is an increasing interest for improving the understanding of collaborative activities among rhizosphere microbial populations and how these might be applied to agriculture^4,5^. The use of microorganisms in agriculture, particularly plant growth promoting rhizobacteria—PGPR-, which efficiently colonize the roots of plants and confer tolerance against several abiotic and biotic stresses^6^ is currently a booming activity. Some of these microorganisms also stand out for their ability to fix nitrogen in a symbiotic or autonomously way, decompose organic waste, detoxify pesticides, suppress plant diseases, provide nutrients to the soil, and produce bioactive compounds such as vitamins and phytohormones that stimulate plant growth^7^.

The search for new strategies for the promotion of plant growth is essential to ensure a safe and sustainable production of vegetables for human consumption^8^. In recent years, the use of associations of microorganisms (consortia) in the field of agrobiotechnology is showing synergistic and more beneficial effects than those applied as single strain. This type of formulation based on microbial co-culture is being promoted^9,10^. A consortium is defined as several species or populations of microorganisms that function in a coordinated and complementary manner so that production, growth and nutrient cycling are improved over what a single species or population can achieve alone under similar environmental conditions^11^. Consortia are model biological systems for understanding the structural and functional requirements of life in extreme environments^11,12^. In fact, the establishment of these associations could increase the chances of microbial survival^13^.

Algae, cyanobacteria and bacteria have coexisted since the early stages of evolution, influencing ecosystems variedly. Several studies have shown that algae and bacteria synergistically affect each other's physiology and metabolism. Hence, there is an urgent need to understand the interactions between autotroph-heterotroph microorganisms and integrate this knowledge for agro-biotecnological use^14^. Cyanobacteria are an indispensable biological component of soil microbiota. Many of them are considered plant growth promoting agents since they are capable to synthesize physiologically active substances stimulating root formation through nitrogen fixation, nutrient mobilization and carbon sequestration in higher plants^15–17^.

Several studies reveal that cell growth and metabolite storage of cyanobacteria (lipids, carbohydrates, pigments, proteins, phytohormones, etc.) can be significantly improved if they are grown together with bacteria considered growth promoters. The remarkable increase in the production of biomass and bioactive compounds from cyanobacteria, as results of co-culture with heterotrophic bacteria, has prompted the study of these mutualistic interactions. Each species shows a particular capability to produce metabolites involved in fertilization, phytostimulation and phytoprotection strategies, so the synergy between different species can derive in obtaining better results when microbial consortia are applied, in relation to the application of axenic cultures^18^. All this leads to the production of more robust crops, capable of better withstanding environmental disturbances^19^. These microbial populations can interact with each other, and exert a positive effect not only on plants, but also on the soil, thereby contributing to the stability and productivity of agricultural systems and natural ecosystems.

Some of those that support photoautotrophic growth are bacteria of agronomic interest. *Pseudomonas putida* is an excellent root colonizer of agronomic relevant crops and it has proven to be effective by improving plant growth and inducing the systemic resistance of plants in response to certain foliar pathogens^20^. In addition, this species stands out for its high capacity to solubilize mineral and organic phosphorus^21^. By exerting these multiple mechanisms, *P. putida* is considered an excellent candidate to the establishment of consortia for agronomic purposes. On the other hand, previous studies have shown the ability of some strains of *Pantoea* spp. to solubilize phosphorus and make it more available for plant uptake^22^. Definitely, microbial consortia often respond to environmental stress as a unique organism, having more chances than any single strain to adapt and take advantage of their internal beneficial interactions^23^.

Despite the potential benefits of formulating microbial consortia from an agronomic point of view, the in vitro ability of cyanobacteria to establish long term or temporary relations with the most ordinary soil bacteria, has not been studied in depth^24^. In this sense, the evaluation of the compatibility and synergism of the microbial components of a consortium is essential to determine its potential use as an agro-biotechnological tool^21,25^. Competition for resources, the cooperation for pollutant abatement, and the ability to act as a biofertilizant or a phytostimulant product will determine the success of a consortium.

Beneficial microbial consortia not only help promote plant growth, but also protect plants from a wide range of direct and indirect environmental stresses. For consortia to be effective, it is imperative that different members positively interact with each other over an extended period of time^26^. A microbial consortium promotes plant growth and health through the production of metabolites with antibiotic activity and by solubilizing nutrients and making them available to the plant, forming nodules to fix nitrogen, and producing plant-growth-stimulating phytohormones or enzymes that degrade ethylene precursors, such as ACC deaminase and suppress plant pathogens^27^.

Therefore, based on the above considerations, the present study focused on the in vitro establishment, evaluation and compatibility study of several two-member consortia (cyanobacterial and heterotrophic rhizobacterial strains) and their subsequent application on tomato plants to estimate their phytostimulant properties at seedling scale.

## Material and methods

### Collection of strains: rhizobacteria and cyanobacteria

In the role of heterotrophic rhizobacteria, two strains were selected from a collection belonging to the BIO175 group of the University of Almería (*Pseudomonas putida*-BIO175 and *Pantoea cypripedii*-BIO175), which stood out for their ability to stimulate plant growth and favor the solubilization of phosphorus and potassium in vitro*,* respectively (Supplementary Table S1). Active pure cultures of both heterotrophic bacteria were kept on Standard Methods Agar (APHA) (ISO 4833:2003) (PanReac Applichem, 413799.1210) at 4 °C until use.

On the other hand, three cyanobacterial strains (SAB-M612 and SAB-B866 belonging to *Nostocaceae* Family) and GS (unidentified cyanobacteria) were selected in terms of their relevance as phytostimulant agents (Supplementary Table S1). The origin of these strains was freshwater, seawater and soil, respectively. Before starting in vitro and in vivo assays, active pure cultures of cyanobacteria were kept on BG11 Agar (BG-11 Broth, SIGMA-ALDRICH, 73816) at 4 °C until use.

### Establishment of consortia

#### Preinoculum preparation

The preinoculum of each cyanobacterium was obtained by inoculating three previously isolated colonies on BG11 agar medium, in a tube with 10 mL of BG11 broth. Incubation conditions were 14 days at 28 °C, 55% humidity, with a photoperiod of 18 h of light and 6 h of darkness, and luminous radiation of 700–1900 Lux (Phytotron, Equitec). After that, 1 mL of the first culture (preinoculum) was distributed among 5 tubes, each one with 10 mL of BG11 broth, and incubated for 7 days under the same conditions previously described. At the end of this time, independent cultures from each cyanobacterium were collected in sterile 100 mL plastic cups. Then, microscopic counts in a Neubauer chamber allowed a determination of the number of microbial cells by direct observation under the microscope. In parallel, a plate count was performed to determine the number of viable microorganisms in our liquid medium. To this end, tenfold serial dilutions in sterile saline solution were performed and 100 μL volumes from dilutions were spread out over Petri plates with BG11 culture media. Counts were expressed as Colony Forming Units per milliliter (CFU ml^−1^).

In the case of the rhizobacteria, isolated colonies previously growing in APHA agar were used as preinoculum and inoculated in a 250 mL flask containing 50 mL of Nutrient Broth. Cultures in this case were incubated at 30 °C, shaking at 120 rpm for 48 h. Likewise, preliminary count of total cells carried out in the Neubauer chamber were followed by plate counts in APHA medium, to determine the number of viable microorganisms, expressed as Colony Forming Units per milliliter (CFU ml^−1^).

The non-phytotoxicity of all the strains used in this work has been previously confirmed through a modified version of the Zucconi test^28^.

#### Establishment of different consortia

This phase of the work consisted of the establishment of consortia between cyanobacteria and heterotrophic rhizobacteria, in two different culture medium, BG11 Broth (BG-11 Broth, SIGMA-ADRICH, 73816) and Algae Broth (SIGMA-ALDRICH, 17124), both prepared in test tubes at a rate of 10 mL per tube. Twelve different experimental blocks (5 axenic cultures, 6 two-member consortia and 1 negative control), from now on treatments, were considered. All treatments were prepared in triplicates. Tubes were inoculated with 1 mL of each microorganism (10% cyanobacteria and 10% heterotrophic bacteria) at doses around 10^5^–10^6^ CFU ml^−1^. All the tubes were incubated in phytotron (Equitec) at 28 °C, photoperiod 18 h of light and 6 h of darkness and 55% humidity.

#### In vitro monitoring of the compatibility of consortia

To evaluate the suitability of the different treatments and the behavior of the two-member consortia in comparison to axenic cultures, microbial counts on BG11 agar and APHA plates were evaluated by the serial dilution method at 24 h, 5 days and 14 days after the establishment of the consortia. APHA plates were kept at 30 °C for three days to count CFU ml^−1^ of heterotrophic bacteria, *Pseudomonas putida*-BIO175 (*P. putida*) and *Pantoea cypripedii*-BIO175 (*P. cypripedii*), while BG11 agar plates were kept at 28 °C for 14 days to count CFU ml^−1^ of cyanobacteria.

### In planta growth promotion

#### Preparation of plant material and microbial inoculum

After finishing the experiment concerning the consortia establishment in vitro, all the treatments evaluated in BG11 broth were used as pre-inoculum to initiate the next experimental phase in vivo*.*

Firstly, a sterile substrate mixture (peat:vermiculite, 3:1 ratio) was prepared, sterilized and spread in seedling trays. Thirty tomato seeds (*Solanum lycopersicum* San Pedro variety) were sown per treatment. Seeds were germinated during three weeks at 25 °C, 60% humidity and a photoperiod of 12 h light and 12 h dark.

Microbial inoculum from each experimental block previously cited was prepared from 20 mL of a growing culture for 14 days in BG11 medium. Preinoculum was added to 250 mL flasks with 180 mL of BG11 broth (1:10 diluted pre-inoculum). Flasks were then incubated in phytotron (Equitec) for 7 days on equal terms described above. After incubation time, inoculum was diluted in tap water (1:10) before root application in tomato seedlings.

#### Inoculation and effects of the consortium in planta

Three weeks after germination, seedling root was irrigated with 25 mL corresponding to each inoculum (microbial suspension). Five randomly placed blocks of six seedlings each were used (a total of 20 repetitions per treatment). A second application was carried out 25 days after the first one in the same conditions. Two weeks after the second irrigation, the following plant growth parameters were measured: stem and root length (cm), stem diameter (mm), fresh and dry weight (g) and root/stem ratio (R/S ratio).

### Statistical analyses

Data obtained were subjected to statistical analysis using Statgraphics Centurion XVII. A multifactorial analysis of variance (ANOVA) and a multiple comparison test (Fisher’s Least Significant Difference) were performed to compare mean values for different levels of repetition (P < 0.05). A linkage between groups was used as a grouping method for the variables analysed in vitro. The interval measured in this case was the nearest euclidean distance squared. Principal Component Analysis (PCA) was used for data reduction and to produce ordination plots. PCA data matrix for plant growth parameters was standardized based on covariances (an eigenvalue = 1 was selected). Finally, Pearson's correlation test was used to assess possible correlations between plant growth parameters respect to treatments with *P. putida* or *P. cypripedii* consortia about axenic cultures. P ≤ 0.05 was required to establish significant differences.

### Policy and plant use guidelines

The authors confirm that the tomato variety (San Pedro) used in the present study was a released variety which is under wide cultivation and was in accordance to international, national, and/or institutional guidelines.

### Statement of permission to use specimens of endangered species

The authors confirm that no any collection of plant or seed specimens was practiced in the present study. The present research does not involve any species at risk of extinction and the convention on the trade in endangered species of wild fauna and flora.

## Results and discussion

### In vitro establishment of consortia

Twelve experimental blocks, were constituted and monitored for 14 days to determine the in vitro behavior of the different microorganisms in axenic or consortium culture, established in BG11 broth and Algae media.

#### In vitro monitoring of heterotrophic bacteria after establishment of consortia

First, growth of heterotrophic rhizobacteria *P. cypripedii* and *P. putida* was determined, both in axenic and mixed culture with 3 cyanobacteria (two-member consortia). The different experimental blocks (1–12), sampling time (24 h, 5 and 14 days), culture media (Algae medium and BG11 broth) and replicates were the variability factors considered to analyze the results of in vitro consortia establishment.

**Figure 1:**
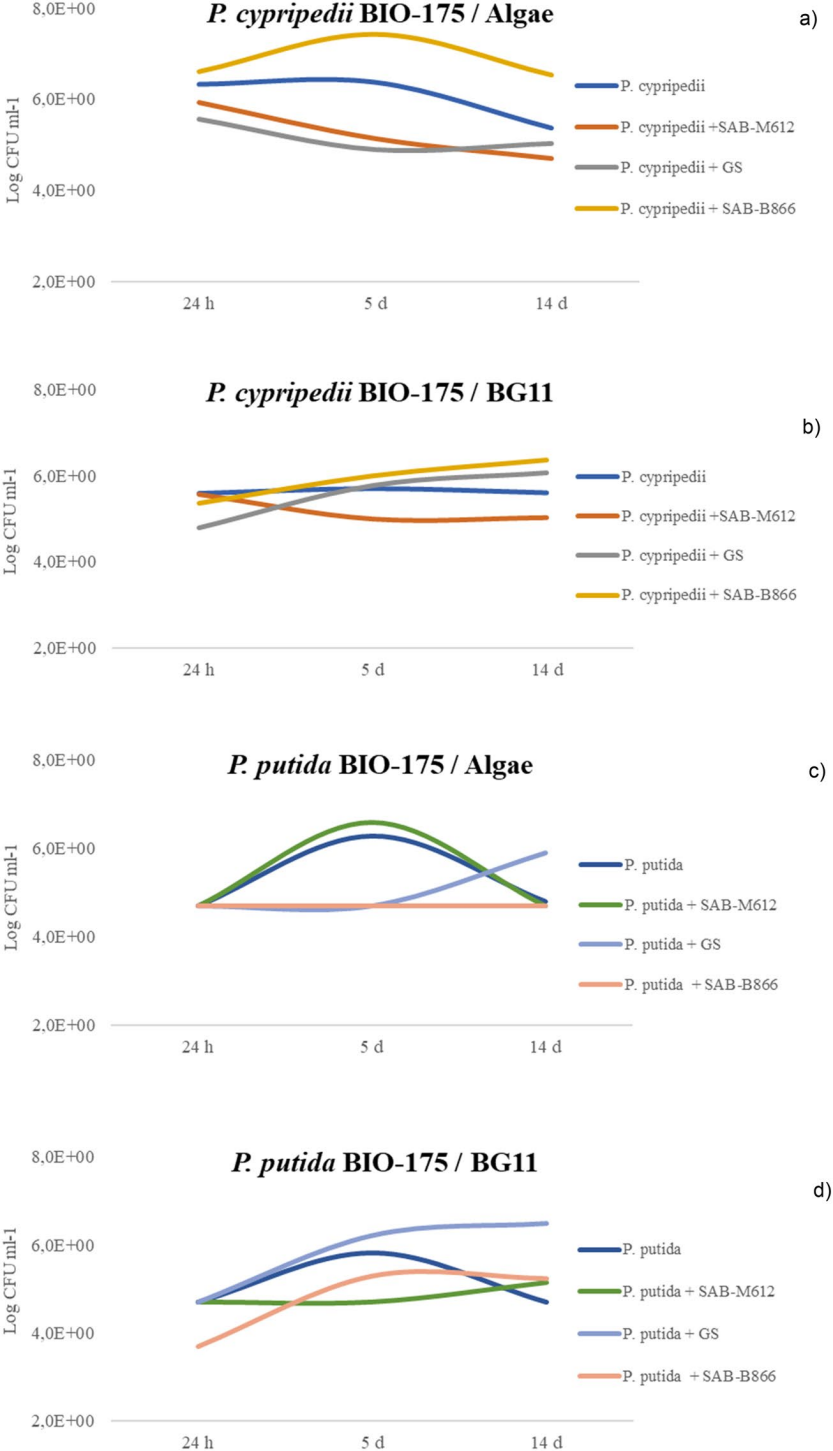
Heterotrophic bacteria counts, *P. cypripedii* (**a**,**b**) and *P. putida* (**c**,**d**). Counts expressed in Log CFU ml^−1^. Evaluated in two culture media BG11 and Algae and at three time intervals: 24 h, 5 days and 14 days.

Figure 1 shows microbial counts in APHA medium graphically represented as Log CFU ml^−1^.

Growth of *P. cypripedii* did not significantly increase in axenic culture, as shown in Fig. 1a,b. However, this trend was reversed in vitro by combining *P. cypripedii* with SAB-B866 and GS, mainly after 5 days from the consortia establishment. Results were variable with respect to cyanobacteria strain and culture medium.

The lowest counts were reported when *P. cypripedii* was cultured in combination with SAB-M612 in BG11 broth (Fig. 1b). In general, *P. cypripedii* strain reduced its viable cell count in mixed culture with SAB-M612 compared to axenic culture. This result could be related to competitive interactions between both species.

The behavior of *P. putida* was similar when it was cultured in both culture media under axenic conditions (Fig. 1c,d), showing maximum count values at 5 days sampling. After this time, microbial count decreased probably due to nutrient limitation and cumulative stress^29,30^. However, the in vitro growth of *P. putida* in consortia depended on culture media and cyanobacterial strain. Thus, *P. putida* growth notably improved in BG11 broth when cultured in combination with SAB-B866 and GS (Fig. 1c,d). On the other hand, consortia with SAB-M612 revealed higher counts in Algae medium than in BG11 broth between *P. putida* and high counts did not increased the *P. putida* growth. Only consortia formed between GS and *P. putida* stand up a more active growth of the heterotrophic bacteria in comparison with microbial counts reported in axenic culture. Maybe the presence of GS seem to have a stabilizing effect on *P. putida*, achieving a steadier growth in co-culture. *P. putida* seem to be more suited for co-cultivation in a photosynthetic consortium as they have lower nutritional demands, as outlined in^29,30^.

**Figure 2:**
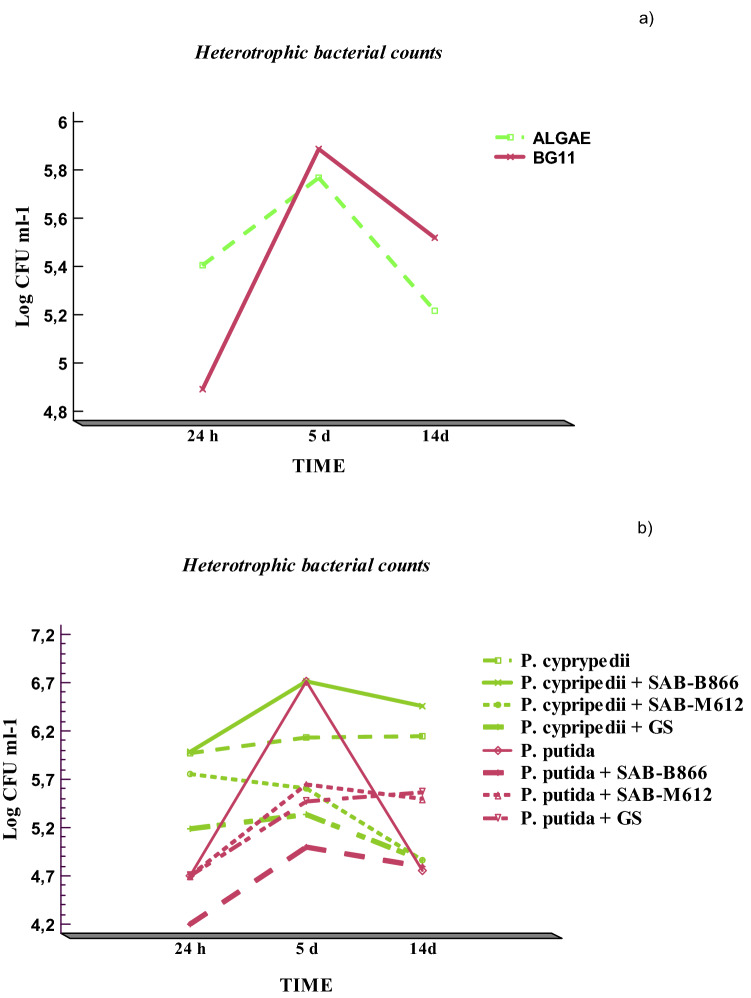
Statistical analysis of the results derived from the heterotrophic bacterial counts: (**a**) bacterial count (Log CFU ml^−1^) with respect to "Time × Culture medium" interaction and (**b**) bacterial count (Log CFU ml^−1^) with respect to "Time × Block" interaction.

Figure 2 shows the results derived from the bacterial count (Log CFU ml^−1^) as a function of the interactions between the variables "Time × Culture Medium" and "Time × Block".

In Fig. 2a, a parallel behavior was observed in both culture media along the incubation time. The highest counts were detected globally at 5 days, and then dropped significantly after that time. In terms of the "Time × Block" interaction (Fig. 2b), it could be confirmed that the highest counts were detected in the consortium formed by "*P. cypripedii* + SAB-B866", with respect to that observed in the control of the *P. cypripedii* strain in axenic culture. By comparison, *P. putida* counts peaked at 5 days of incubation in axenic culture, but then declined sharply. However, although with lower counts, the stationary phase of *P. putida* was maintained until 14 days, when it was cultured in consortia with the cyanobacteria (Fig. 2b).

Microbial consortia are established in nature on the basis of metabolic relationships that are beneficial to the various parties involved. In view of the results derived from this first phase of the work, it is clear that, when formulating a mixed microbial inoculum, it is necessary to evaluate whether the optimal establishment of the consortium in vitro takes place, taking into account the compatibility of their members as well as the most suitable conditions for its cultivation^31^. These results suggest that heterotrophic bacteria in co-culture with cyanobacteria could take advantage of several components secreted or released from the phototrophic organism. In addition, cyanobacteria could help maintain the aerobic environment of the consortium as well as being providers of organic carbon^32^.

The different behaviors observed when combining phototrophs and heterotrophs strains in co-culture reveal that the symbiotic relationship depends on the specific species, which has been previously reported. Several authors suggested multiple factors that favor the relationships between consortia members: (1) accessible organic carbon provided by the phototroph to the heterotroph; (2) similar desirable cultivation conditions for the partners and, finally, (3) absence of toxic or lethal factors produced by one of the involved organisms^32,33^ showed that the supernatant of different strains of cyanobacteria belonging to the genus *Nostoc* spp. favored the axenic growth of fungi thanks to the presence of proteins and polysaccharides, which serve as energy and carbon supply for fungi. In the same line, previous research has demonstrated that the presence of heterotrophs can enhance the growth of phototrophs. Photoautotrophic members, cyanobacteria or eukaryotic algae, convert CO_2_ into organic carbon for the heterotrophic growth of their microbial partner. Exchange of these metabolites can sustain the heterotrophs under conditions of absence of any organic carbon source. In turn, the heterotrophs provide additional CO_2_, protection against environmental factors and often a diverse array of secondary metabolites^34^.

#### In vitro monitoring of cyanobacteria after establishment of consortia

**Figure 3:**
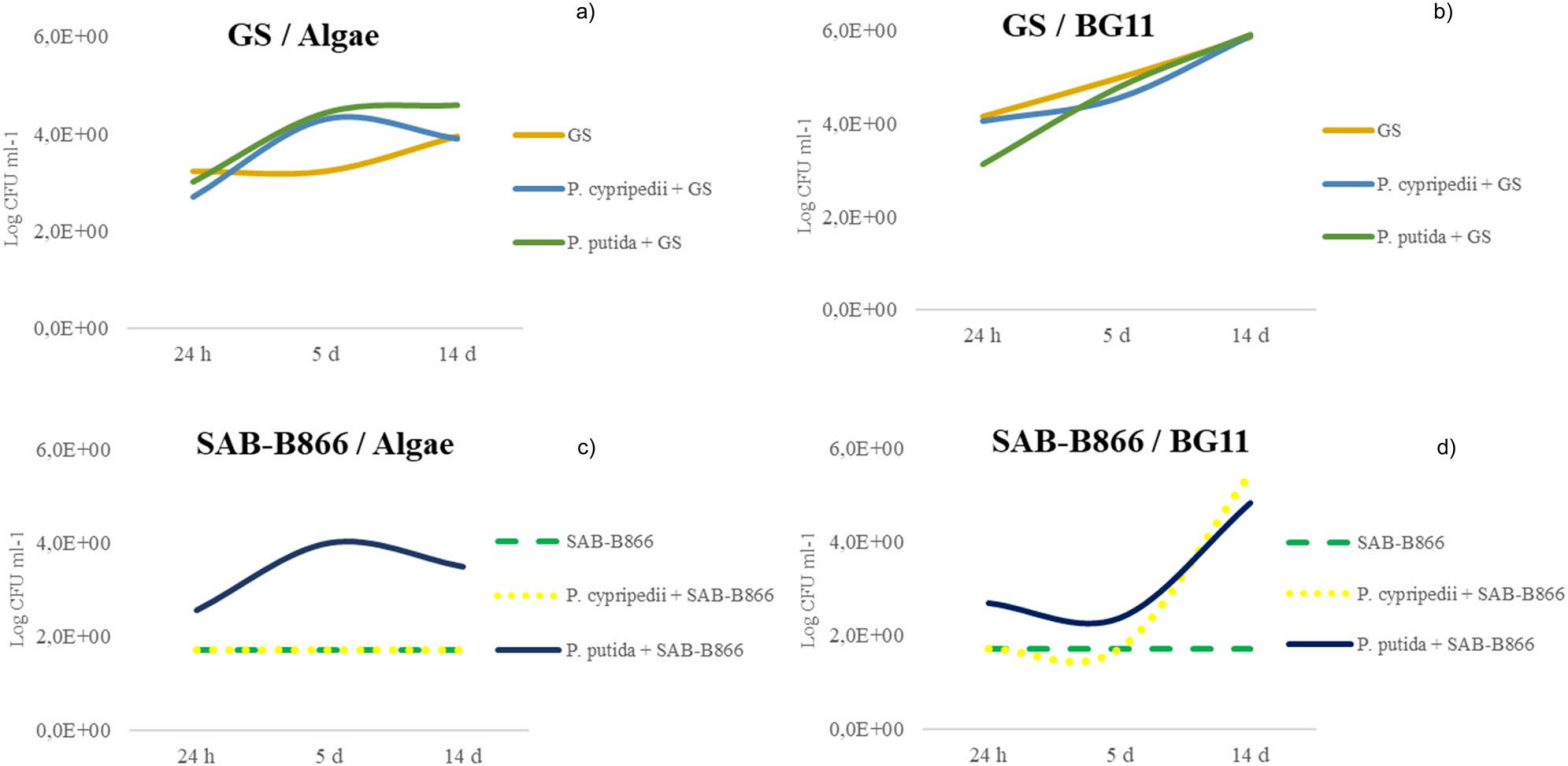
Cyanobacteria counts, GS (**a**,**b**) and SAB-B866 (**c**,**d**). Counts expressed in Log CFU ml^−1^. Evaluated in two culture media BG11 and Algae and at three time intervals: 24 h, 5 days and 14 days.

Growth of cyanobacteria participating in the consortia with both heterotrophic bacteria was also evaluated. Figure 3 shows microbial counts (Log CFU ml^−1^) in BG11 broth and Algae media.

SAB-M612 did not increase the number of CFU ml^−1^ in axenic culture (results not shown). This allows understanding why in the consortia established between SAB-M612 and the rhizobacteria, *P. cypripedii* and *P. putida,* a completely satisfactory behavior was not detected. Probably, the culture conditions were not optimal for the growth of the cyanobacteria, consequently affecting the growth of the rhizobacterial.

On the other hand, GS showed the best behavior, both in axenic culture and consortia (Fig. 3a,b). Counts of GS in BG11 broth were in general higher than those observed in Algae medium. However, there was not difference among axenic and consortium culture when GS was cultured in BG11 (Fig. 3b). In contrast, the growth rate of GS using Algae medium was faster in consortium (Fig. 3a).

In turn, SAB-B866 counts showed a remarkable increase in both culture media after the interaction with both heterotrophic bacteria (Fig. 3c,d). However, it should be noted that its association with *P. putida* favored the population increase in both culture media, while its growth in consortia with *P. cypripedii* was only favored in Algae medium.

In view of the results obtained, heterotrophic bacteria could stimulate the cyanobacterial growth when they are members of the same consortia. In fact, it has been postulated, according to some authors, that cyanobacteria are dependent on other aerobic heterotrophic bacteria for their growth, which explains the difficulty of some of them to grow and maintain viability in axenic culture^35^. The results derived from this work partly confirm this dependence, especially in the case of strain SAB-B866, showing a population increase extremely dependent of culture media and accompanying partner.

**Figure 4:**
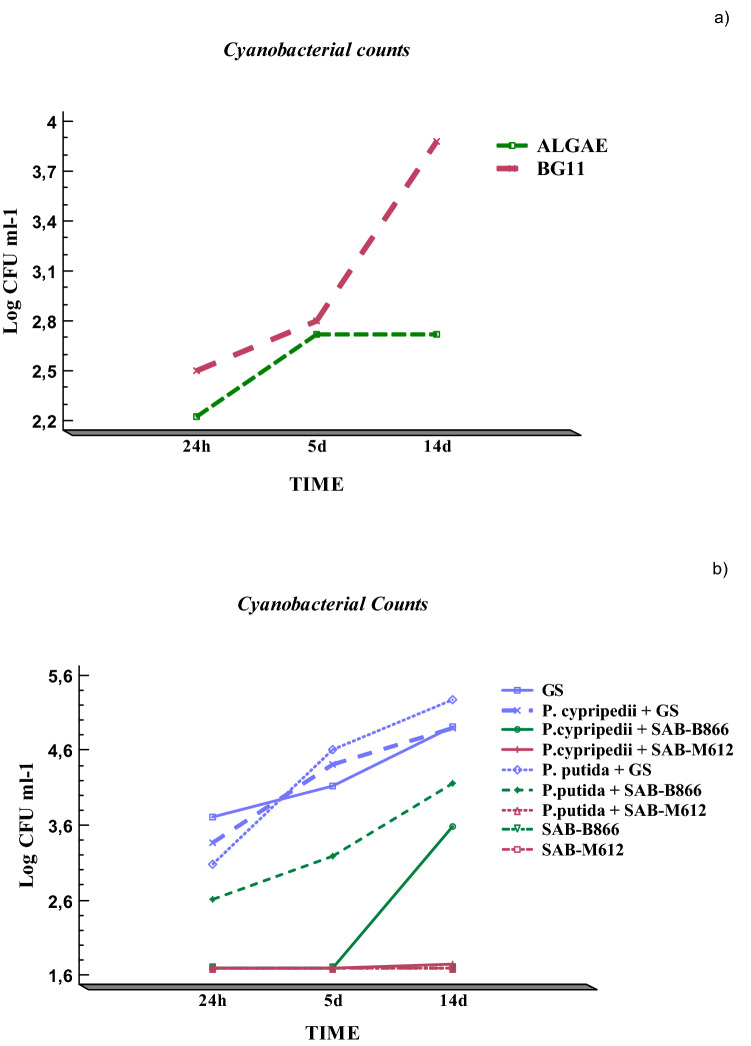
Interaction analysis between variability factors: (**a**) Cyanobacterial counts (Log CFU ml^−1^) with respect to "Time × Culture medium" interaction and (**b**) Cyanobacterial counts (Log CFU ml^−1^) with respect to "Time × Block" interaction.

Figure 4 shows the results derived from the cyanobacterial counts (Log CFU ml^−1^) as a function of the interactions between the different factors analyzed ("Time × Culture Medium" and "Time × Block").

During the first 5 days of the establishment of the consortia, the culture medium did not significantly affect the cyanobacterial counts obtained (Fig. 4a). From that moment on, there was an increase in the cyanobacterial counts mainly in BG11 broth, with respect to those obtained in Algae medium. It was noticeable that the counts obtained for the cyanobacterium SAB-B866 in consortium with each heterotrophic bacteria were generally higher than those observed in axenic culture (Fig. 4b). On the other hand, the consortium between the GS and *P. putida* was not only compatible, but also implies an increase in the growth of the cyanobacterial partner. Regarding SAB-M612, satisfactory results were not reached in general terms, since no significant population increase was detected in any of the experimental blocks analyzed, as indicated above.

According to Hays et al.^36^, the growth of all heterotrophic species could stimulate the growth of cyanobacteria. However, some authors defend that a positive cooperation only are produced if both members show a lower cell density in axenic culture in comparison to detected in mixed culture. In this way, microbial cooperation would entail that the total number of cells in dual culture be higher than the sum of the monocultures^37^. In contrast, other combinations are characterized by mutual inhibition, with a decrease in cell numbers of both strains in comparison to checked in axenic culture. In view of the results described in this first part of the work, *P. putida* address to growth promotion of the cyanobacteria GS and SAB-B866. *P. putida* is classified as a colonizer rhizobacteria of a wide range of plants. It can promote the plant growth through nitrogen fixation, phosphate solubilization, or production of phytohormones, such as gibberellin and indol-3-acetic acid^38,39^. Many of these properties could notably favor the growth of cyanobacterial member of the consortia.

### In planta growth promotion

#### Evaluation of the application of the different experimental blocks containing *P. cypripedii* on tomato seedlings

**Figure 5:**
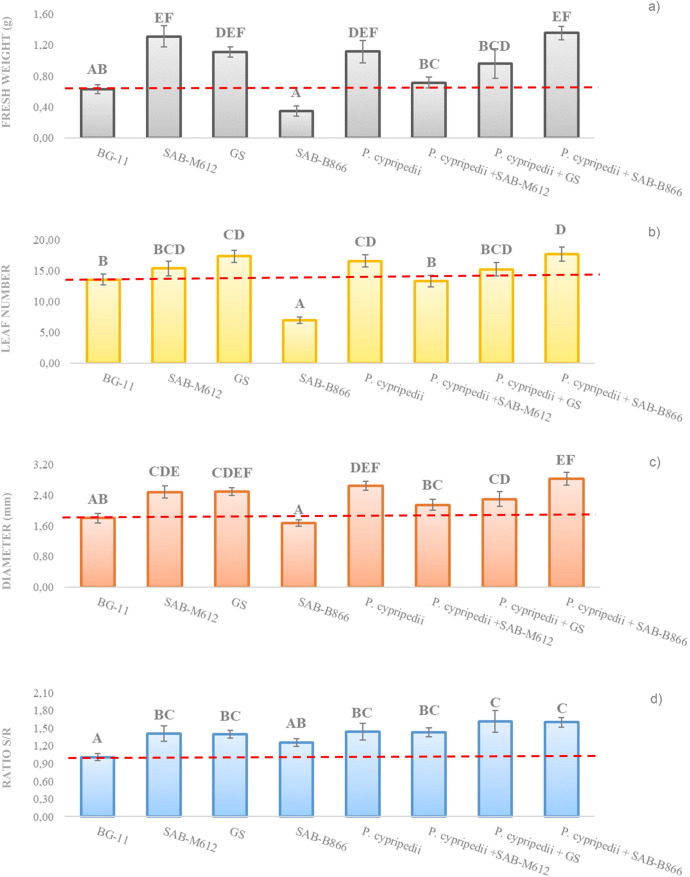
Evaluation of different growth parameters in tomato seedlings, after treatment with experimental blocks containing *P. cypripedii*. Representing (**a**) fresh weight (g), (**b**) number of leaves, (**c**) stem diameter (mm) and (**d**) stem:root ratio. The vertical bars represent the standard error values.

The effect of the application of the different experimental blocks containing *P. cypripedii* was evaluated on tomato seedlings by determining seven basic growth parameters such as: stem and root length, leaf number, stem diameter, stem:root ratio, fresh and dry weight. The most relevant results of the in vivo assay are shown in Fig. 5a–d.

In general terms, treatment factor significantly affected growth parameters outperforming the negative control (BG11) in any of the parameters analyzed (except for the treatment with the cyanobacterium SAB-B866 in axenic culture). It is worth mentioning the increase observed in fresh weight (Fig. 5a), leaf number (Fig. 5b), stem diameter (Fig. 5c) when application of the consortia established between *P. cypripedii* and the cyanobacterium SAB-B866 (a modest but very noticeable increase in relation to the application of cyanobacteria in monoculture). Regarding stem:root ratio (Fig. 5d), the consortia established between *P. cypripedii* with the cyanobacteria GS and SAB-B866, overcame the effect caused by the rest of the treatments applied in pure culture, which resulted in better stem development with both treatments.

#### Evaluation of the application of the different experimental blocks containing *P. putida* on tomato seedlings

**Figure 6:**
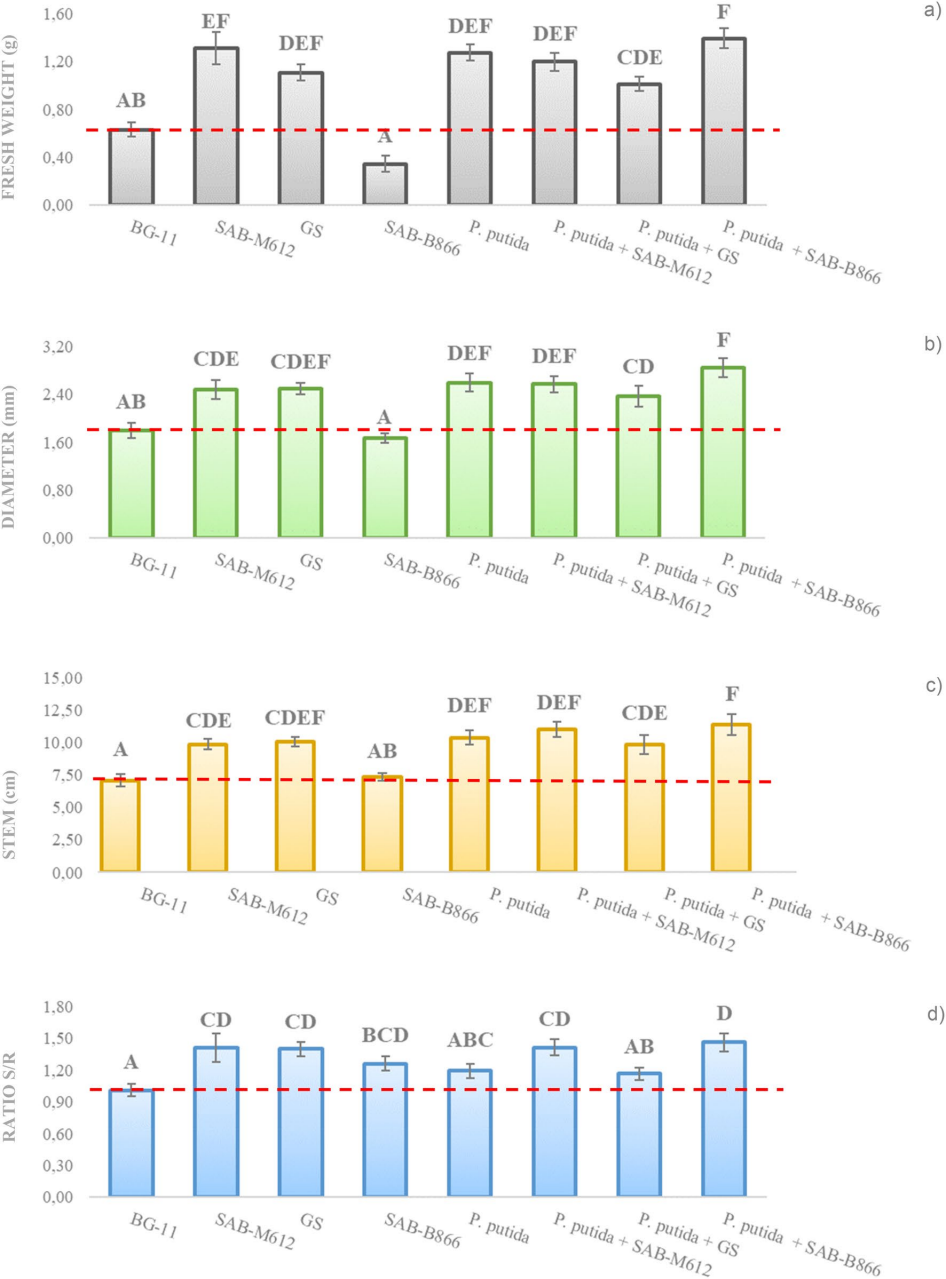
Evaluation of different growth parameters in tomato seedlings, after treatment with experimental blocks containing *P. putida*. Representing (**a**) fresh weight (g), (**b**) stem diameter (mm), (**c**) stem length (cm), and (**d**) stem:root ratio are represented. Vertical bars represent standard error values.

As indicated in Fig. 6, interesting behavior was observed after the treatment with *P. putida* in consortium with the cyanobacteria SAB-B866.

All growth parameters on tomato seedlings were increasing in relation to control plants (BG11), excepting for root length (Figure not shown). On the other hand, consortium between *P. putida* and SAB-M612 showed also relevant results in relation to fresh weight, diameter and stem length (Fig. 6a–c). The differences between treatments were also evident in the estimated index, stem:root ratio (T/R), where the lowest value expressed was for the plants inoculated with BG11, while the rest of the treatments favored stem development, highlighting the values obtained with the combinations *P. putida* with the cyanobacteria SAB-M612 and SAB-B866 (Fig. 6d).

Microbial inoculants are promising components of sustainable crop systems because of their capacity to promote plant growth, enhance nutrient availability and uptake, and support the health of plants^40^. This has been especially reported especially in the case of rhizobacteria interacting with tomato crops^41,42^, as observed in this work. Biofertilizers show no detrimental effects on crops. In addition, they could be applied directly to the soil, seeds, or foliar surface. They also increase the level of nutrients in plants and allow them to grow in a healthy environment^43^. Our study has shown beneficial effects of some microbial consortia in relation to axenic cultures. The established consortia had shown a slight increase of the plant height, diameter and fresh weight of tomato. This may be due to the synergistic ability of the selected strains. According to Stockwell et al.^44^, microbial consortia may also improve their efficacy, reliability and consistency under different factors such as light intensity, pH, substrate and temperature^45^, as well as compatibility between associated strains^46^. Earlier studies using cyanobacteria^47^ revealed their significant role on *Solanaceae* and *Cucurbitaceae* crops in terms of root and stem length, leaf number, stem diameter, fresh and dry weight. According to Rai et al.^48^, microbial consortia contribute to plant development by either direct or indirect fixed-N transfer, improving soil quality and nutrient status, solubilizing inorganic phosphates, as well as producing hormones and vitamins. In addition, the ability to produce synergistic effects related to plant growth by combining different PGPMs (Plant Growth Promoting Microorganisms), beyond the effect observed after individual application, has been proven^49,50^.

Choosing the right microbial consortium is essential. To assess its potential there are direct observable factors like nutrient uptake, oxygen levels, or biomass growth. Additionally, exist the possibility to lump the metabolic pathways of several species together, obtaining a consortium where the biochemical reactions are acting together to optimize a common objective function. Another approach is to build a compartmentalized model, where each species is a separate compartment and transport fluxes allow the exchange of metabolites between them. The soil is the main affected by the microbial community associated with this ecosystem, therefore, the roots of the plants are in the first instance affected by the changes that occur in it. However, this has a significant effect on the rest of the plant. In short, the new lines of study will focus on the analysis of crop exudates to assess the molecules that plants use to communicate with soil microorganisms, as well as the pathways they use to decipher these signals, which in turn helps to understand the interaction among the microbial partners of future consortium formulations^51^.

#### Statistical analyses of the results derived from in vivo bioassays

**Figure 7:**
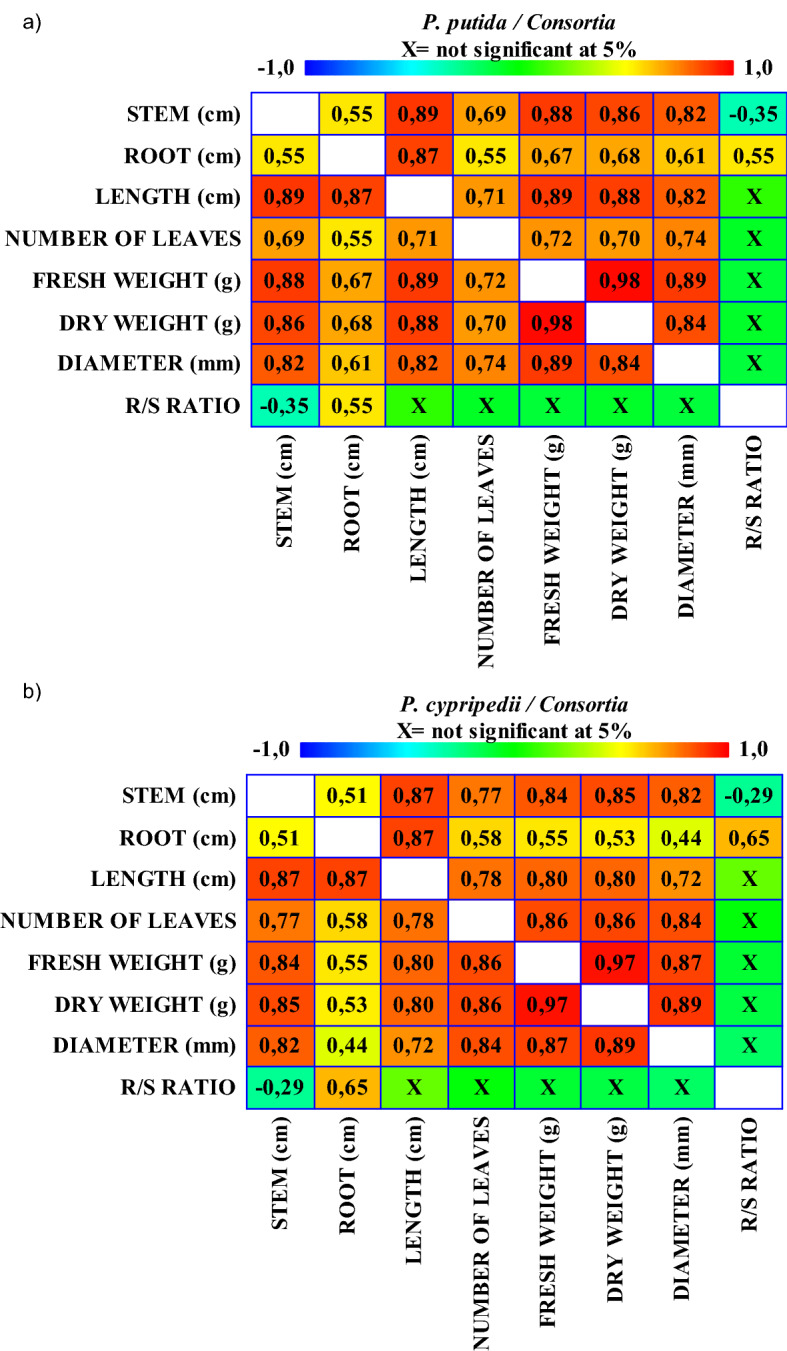
Pearson correlation analyses of the results derived from in vivo bioassays, at 95% confidence level, *P. putida* (**a**) and *P. cypripedii* (**b**). Plant growth parameters considered: Stem (cm), Root (cm), Number of leaves, Stem Diameter (mm), Fresh weight (g) and Dry weight (g).

In Fig. 7, a Pearson correlation analyses was carried out in relation to the plant growth parameters analyzed in this work, which were stem and root length, leaf number, stem diameter, root:stem ratio, fresh and dry weight.

Pearson R value oscillated between − 1 and + 1, as negative relationships between variables were represented in blue, while positive ones were indicated in red. Figure 7a shows all data referring to the experimental blocks containing *P. putida* and Fig. 7b shows those data concerning to treatments with *P. cypripedii* blocks. As expected, the root:stem ratio correlated negatively with stem length measurement, while it correlated positively with root length. However, strong positive correlations were also established between stem length and other parameters related to aerial plant growth. This fact may be indicative that the treatments applied widely stimulated the size of the plant (vegetative growth). Figure 7 shows the greatest similarity in the behavior of tomato seedlings when treated with the experimental blocks including *P. putida* (Fig. 7a) or *P. cypripedii* (Fig. 7b) (in axenic culture or in consortia with cyanobacteria). In general, data have proved that aerial growth stimulation occurs in general contrary to root development.

On the other hand, the relationship among the variables in vivo evaluated was analyzed by a Principal Component Analysis (PCA) (Supplementary Fig. S1).

Establishing two principal components (PCs), the model explained more than 90% of the data variability, with the following contributions of each: PC1 70.97% and PC2 18.60%, when the seedlings were inoculated with the experimental blocks containing the rhizobacteria *P. putida* (Supplementary Fig. S1a); PC1 70.20% and PC2 20.83%, when the seedlings were inoculated with the experimental blocks containing the rhizobacteria *P. cypripedii* (Supplementary Fig. S1b). In both cases, PC1 was mainly associated with parameters of the vegetative part of the plant, while PC2 was directly associated with plant root development. Regarding PC1 showed a close positive associations with the variables concerning the aerial growth such as stem length, leaf number, fresh and dry weight and diameter, while PC2 component revealed a strong positive association with root:stem ratio and root length.

Supplementary Fig. S1 shows the greatest similarity in the behavior of tomato seedlings when treated with the experimental blocks including *P. putida* (Supplementary Fig. S1a) or *P. cypripedii* (Supplementary Fig. S1b) (in axenic culture or in consortia with cyanobacteria). In general, data have proved that aerial growth stimulation occurs in general contrary to root development.

The relationship between cyanobacteria and bacteria is greatly dependent on the species and the environmental conditions. In the beneficial relationship, cyanobacteria enhance bacterial growth by providing photosynthetic oxygen and dissolved organic matter^52^. In turn, bacteria support photoautotrophic growth by providing carbon dioxide and other stimulatory means^9^. Actually, both partners can produce plant growth promote biomolecules^53,54^. Recently, Horácio et al.^55^ evaluated the effects of co-inoculation with cyanobacteria and bacteria, getting an increase in nodulation, plant growth and production of the common bean, comparing with the untreated controls. This effect is indicative that N fertilization could be replaced by the use of previously selected microbial consortia.

The results derived from the in vivo evaluation of the different experimental blocks corroborated that observed in other previous works. Ibiene et al.^56^ analyzed the synergistic effect of different consortia formed by a combination of three rhizobacterial strains on tomato seedlings. They evaluated the total length and stem diameter in treated plants with the strains in pure culture or in consortia. The results obtained were very enlightening, since the use of consortia proved to be more successful than the application with the strains in axenic culture. Other more recent studies, focused on the evaluation of germination rates, stem length and diameter, or dry weight, among others, have clearly shown the synergistic or additive effect of treatment with microbial consortia compared to the independent inoculation of the same microorganisms^57,58^.

Since no production studies have been carried out, it is not possible to know how adult plants would have behaved after treatment with the different consortia; a priori, an increase in the aerial part of the plant (diameter, stem length, number of leaves) could be expected. On the other hand, the dosage of microorganisms used for the establishment of the consortia, or for seedling inoculation, was unique. Therefore, the search for the best possible combinations, the optimal in vitro or in vivo doses, or the most appropriate phenological stage to carry out the application of this type of "bioproducts” in an integrated manner, is part of future experimental designs.

In brief, the results showed that the consortia application on cucumber seedlings produced an increase in the growth, and it shows the role of the consortia as promoter of plant growth in cucumber crops. Although growth promotion has not been specifically contrasted with analytical tests to evaluate the nutritional status of the plant, prior knowledge about both rhizobacterial strains, *P. putida* and *P. cypripedii*, helps to draw important conclusions. This fact could directly be related to its demonstrated abilities to solubilize phosphorus and potassium (Table S1), by producing solubilization halos in the specific culture media for detecting potassium and phosphorus solubilization^59^, respectively. Since their application in plant derived in a remarkable elongation of the stem, which could derive from the greater availability of phosphorus to the plant, since as described by some authors a deficit of this element can cause restrictions in the growth of the stem^60^. On the other hand, its application globally caused a significant root elongation, probably due to the increased availability of potassium to the plant. Some authors describe that a potassium deficit can lead to a poor root system and stem weakness^61^.

## Conclusion

In this work, the positive behavior of some heterotrophic rhizobacteria in consortia with cyanobacteria from different source, show the enormous metabolic versatility and adaptability of this type of microorganisms. In view of the results obtained, the establishment of microbial two-member consortia (heterotrophic rhizobacteria and cyanobacteria) does not always occur successfully. This association depends in depth on the culture medium, incubation time and microbial components of the consortium. In this work, while SAB-B866 and GS cyanobacteria showed a population increasing in presence of both rhizobacteria, the same did not occur with SAB-M612. As for the in vivo treatment, the consortia established between the cyanobacteria SAB-B866 and the heterotrophic rhizobacteria *Pantoea cypripedii* and *Pseudomonas putida*, gave very satisfactory results compared to those obtained from treatment with the SAB-B866 as independently. Consortia between the cyanobacterium SAB-B866 and both heterotrophic rhizobacteria are strongly compatibles, such as demonstrating the increase in the microbial counts mainly in BG11 medium, with respect to those obtained in Algae medium. In addition, the establishment of these consortia implies an increase in the aerial growth of tomato seedlings, outperformed the control block. It is worth noting the proliferation of the stem, as well as the fresh weight and diameter.

In short, the versatility and plasticity, as well as the robustness of the microbial communities involved in this type of consortia, describes the potential of these microbial communities to be applied as new agro-biotechnological strategies. However, the ability of the strains to control plant pathogens will be addressed in other subsequent works, more related to phytoprotective activity of microbial strains.

## Supplementary Information

Supplementary Table S1.

Supplementary Figure S1.

## Data Availability

The datasets generated during and/or analysed during the current study are available from the corresponding author on reasonable request.
