# Supplementary material for: Design and validation of cyanobacteria-rhizobacteria consortia for tomato seedlings growth promotion
Source: Sci Rep. 2022 Jul 31;12:13150. doi: 10.1038/s41598-022-17547-8 (PMC9339543; doi:10.1038/s41598-022-17547-8)
Supplement: Supplementary file 1 — Supplementary Table S1. [file 41598_2022_17547_MOESM1_ESM.docx]

**Supplementary Table S1**. Strain characterization as potential phytostimulant agents

| Strain | Germination Index (%)^1^ | Phosphate solubilization^2^ |  | Potassium solubilization^3^ | Salycilic acid producer^4^ | Siderophore producer^5^ | Stimulation of seedling growth |
| --- | --- | --- | --- | --- | --- | --- | --- |
| *Pseudomonas putida*-BIO175 | 80-90% | + |  | + | + | + | + |
| *Pantoea cypripedii*-BIO175 | 94% | + |  | + | n.t | n.t. | + |
| *Nostoc* SAB-M612 | > 100% | - |  | n.t | + | - | + |
| *Dolichospermum* SAB-B866 | 90-100 % | - |  | - | - | - | + |
| GS (unidentified cyanobacterium) | 80-90% | - |  | - | n.t | - | + |

^1^[28].

^2^Pikovskaya medium [62].

^3^Aleksandrow medium [63].

^4^Plant Salicylic acid SA ELISA Kit, MyBiosource MBS9314138.

^5^CAS medium [64].

n.t.: non tested
