# Supplementary material for: Design and validation of cyanobacteria-rhizobacteria consortia for tomato seedlings growth promotion
Source: Sci Rep. 2022 Jul 31;12:13150. doi: 10.1038/s41598-022-17547-8 (PMC9339543; doi:10.1038/s41598-022-17547-8)
Supplement: Supplementary file 2 — Supplementary Figure S1. [file 41598_2022_17547_MOESM2_ESM.docx]

**Supplementary Fig. S1**. Results of principal component analysis (PCA) based on measurement of plant growth parameters from tomato seedlings in relation with root application with generated experimental consortia containing *P. putida* (a) or *P. cypripedii* (b). All results are means (n=20 repetitions).

b)

a)
